# Feasibility of parathyroid gland autofluorescence imaging after indocyanine green fluorescence angiography

**DOI:** 10.3389/fendo.2023.1248449

**Published:** 2023-11-03

**Authors:** Marieke Richard, Philippe Rizo

**Affiliations:** Fluoptics SAS, Grenoble, France

**Keywords:** parathyroid autofluorescence, ICG fluorescence angiography, NIR intraoperative fluorescence imaging, thyroid Surgery, hypocalcemia, hypoparathyroidism

## Abstract

**Background:**

In thyroid surgery, autofluorescence allows the parathyroid glands (PTGs) to be located very early to protect them. Moreover, indocyanine green (ICG) fluorescence angiography (ICG-FA) allows for assessing the viability of the PTGs and identifying blood vessels to preserve them. The main limitation of using ICG-FA is that once ICG has been injected, it is no longer possible to observe PTG autofluorescence using existing devices. This study aimed to describe an approach that allows for visualization of the PTGs by autofluorescence, even after ICG injection.

**Methods:**

We redesigned the FLUOBEAM® LX system to excite fluorescence at 685 nm and detect fluorescence between 700 and 900 nm. This device had short-pass filters at 775 nm that helped to split the contributions of the PTG autofluorescence and ICG fluorescence. Tests were performed on extemporaneous PTG preparations placed next to ICG droplets to assess for rejection of the ICG signal.

**Results:**

A low-pass filter at 775 nm detected 60% of the autofluorescence signals and 10% of the ICG signals.

**Conclusion:**

These findings support the possibility of visualizing PTG autofluorescence despite multiple ICG injections and measuring the balance between ICG and autofluorescence signals.

## Introduction

The preservation of the parathyroid glands (PTGs) and their vascularization is crucial during thyroidectomy because damage to the PTG can result in postoperative hypoparathyroidism and hypocalcemia, which can significantly increase cardiovascular and renal morbidity and patient mortality (1, 2).

Autofluorescence has recently been introduced in thyroid surgery to facilitate the detection of PTGs (3–6). Initially, surgeons relied only on their experience and training to detect the parathyroid glands and decide which blood vessels to preserve to protect gland vascularization during thyroidectomies. Depending on the surgeon, in 30–60% of cases, autofluorescence imaging allows for the detection of the PTGs before the surgeon sees them. Although, in some cases, autofluorescence allows for better preservation of the PTG (4), it does not provide any information regarding the vascularization pathway of the glands. Identifying the blood vessels feeding the PTGs is critical because the vascularization of the PTG is unpredictable (7). Recently, fluorescence angiography using indocyanine green (ICG) during thyroidectomy has demonstrated the possibility of identifying critical blood vessels needing preservation (8). 0.1mg/kg of ICG is usually intravenously injected as a bolus. 10 to 30 seconds after injection, ICG reaches the thyroid gland and fluorescence signal can be detected by the imaging device and displayed on a screen.

The main limitation of ICG fluorescence angiography, used for detection in the range of 800–900 nm, is that once ICG has been injected, even at 0.1mg/kg, it would no longer be possible to visualize the autofluorescence of the PTGs. ICG Angiography is performed to visualize small blood vessels. Therefore, injected dose leads to high levels of fluorescence that overwrite parathyroid autofluorescence. ICG fluorescence is at least one order of magnitude brighter than PTG autofluorescence in the range of 800–900 nm. Currently, available systems use fluorescence excitation wavelengths longer than 740 nm and detect in the range 800-900nm (9). Thus, once ICG is present in the field, the PTG autofluorescence signal becomes negligible compared with the ICG signal.

Thyroidectomies are usually performed in two phases (one lobectomy per side), and the surgeon must preserve the PTGs independently on each side. If the surgeon wants to use autofluorescence on both sides of the thyroid, then ICG can only be used on the second operated side. Consequently, during total thyroidectomy using existing devices, ICG fluorescence angiography can be combined with autofluorescence on only one side of the patient.

With classical devices based on excitation wavelengths longer than 740 nm, a waiting time of about 1 hour is required between the last ICG injection and the next autofluorescence measurement. However, despite this delay, several areas remain marked by ICG because it is injected into the dissected tissue, which degrades the readability of the autofluorescence images by registering many ICG-related false positives.

Furthermore, the inability to use perfusion imaging on the two operative sides leads to (i) a potentially greater risk of damaging the vascularization of the PTGs of the first lobe and (ii) not knowing if the PTGs left in place on the first lobe are functional. This has a significant implication on how the second lobe should be dissected, especially if the PTGs on the second lobe are poorly localized. Therefore, it is crucial to visualize the autofluorescence of the PTGs *in situ* even after ICG injection. Here, we described an approach that allows for visualization of the PTGs by autofluorescence, even after ICG injection.

## Materials and methods

ICG (25 mg) was purchased from Diagnostic Green (Indocyanine Green for Injection Kit, 25 mg). Bovine Serum Albumin was purchased from Sigma Aldrich (Bovine Serum Albumin, 5%; reference A4628-20ML). We performed fluorescence spectrometry measurements of ICG diluted with albumin at an excitation wavelength of 685 nm. We observed that the fluorescence yield was 30–50% lower than that measured at an excitation wavelength of 750 nm. The detection was performed on a larger bandwidth of 700–900 nm. The maximum fluorescence of ICG excited at a wavelength of 685 nm was observed near 820 nm.

**Figure 1:**
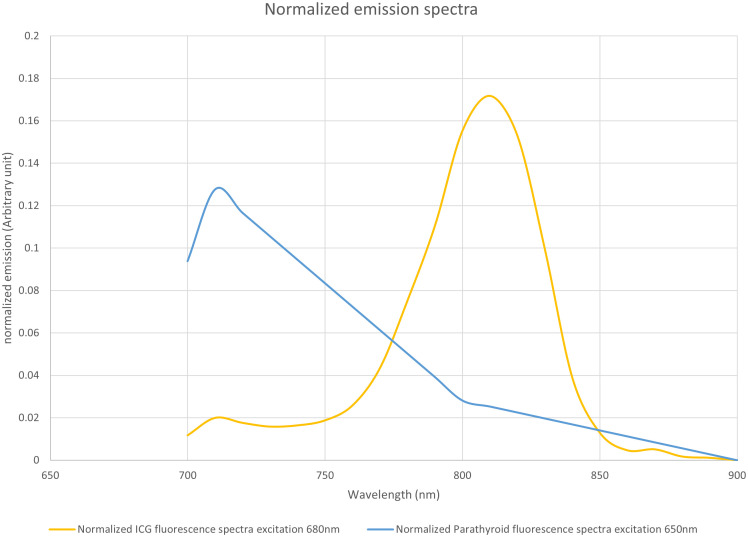
Normalized emission spectra of indocyanine green (ICG) when excited at 685 nm and parathyroid gland (PTG) autofluorescence when excited at 650 nm. Both spectra were plotted with an arbitrary unit such that the area under the curve equaled 1. The fluorescence units are, therefore, different between ICG and PTG autofluorescence. However, the maximum emission peaks were adequately separated. The data for the PTG spectrum was derived from the study by Serra et al. (10), while that for the ICG was measured on a Perkin Elmer LS50B system. ICG (25 mg) was solubilized in sterile water (10 mL). Further, the stock solution was diluted with bovine serum albumin to obtain a 1 µM solution. This diluted solution was placed in a Polymethyl Methacrylate cuvette, and the ICG emission spectrum was measured with 685 nm excitation. We assumed that the autofluorescence spectra for wavelengths longer than 700 nm were similar, with an excitation wavelength at 650 nm and 685 nm, respectively.

Next, a comparison of the emission spectra of PTG autofluorescence for excitation at 650 nm, as provided by Serra (10), with that of ICG (**Figure 1**) showed that their peak maxima were well separated. We assumed that the autofluorescence spectra for wavelengths longer than 700 nm were similar, with excitation wavelengths of 650 and 685 nm.

To alternatively visualize PTG autofluorescence and the ICG signal on the same system, we modified a FLUOBEAM_®_ LX device (FLUOPTICS^©^ France) by replacing the 750 nm excitation laser with a 685 nm laser and also fit detection filters with a range of 700–900 nm. This system will hereafter be referred to as the LX700. The excitation power at the area of interest was 4.6 mW/cm^2^ at a distance of 10 cm from the optical head, and we ensured that the device was operating in laser class 1 (11).

**Figure 2:**
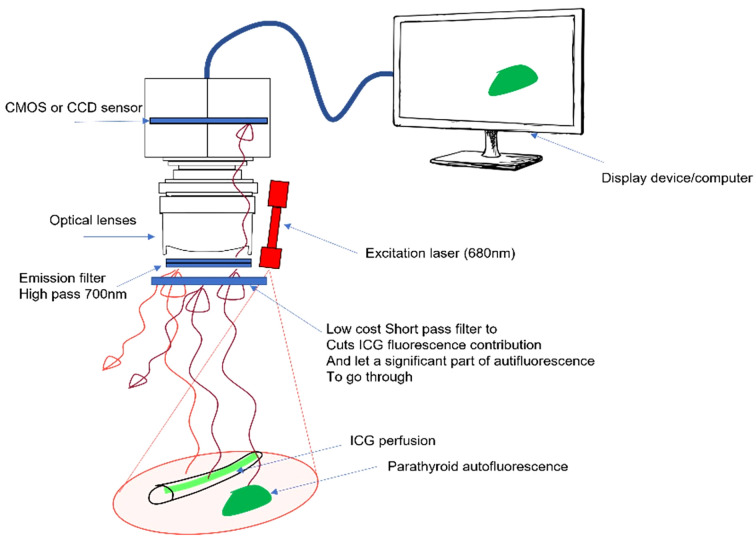
Proposed approach with a removable low-pass filter. The device excites autofluorescence of the parathyroid gland (PTG) and indocyanine green (ICG) fluorescence at 685 nm and reads the signal between 700 and 900 nm. A removable low-pass filter is placed in front of the camera sensor to narrow the detection window between 700 and 775 nm, the goal being to manage the ratio between the ICG and PTG signals.

To separate the PTG autofluorescence signal from the ICG fluorescence signal using this device, we tested a retractable low-pass filter with a cut-off wavelength of 775 nm in front of the camera sensor. This led to two spectral detection windows: 700–900 nm without the filter and 700–775 nm with the filter. The experimental setup is shown in **Figure 2**.

**Figure 3:**
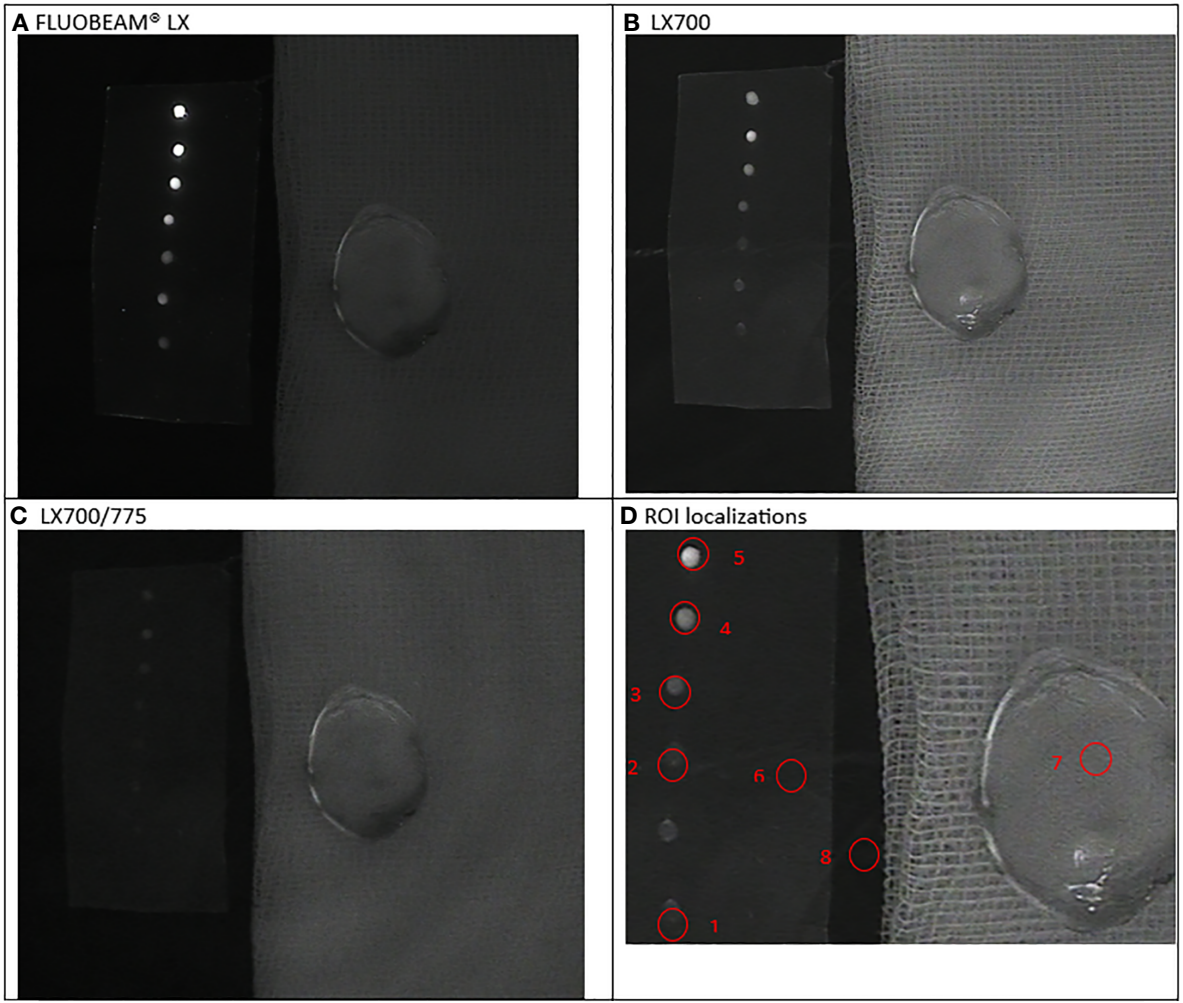
First experimental setup: Parathyroid adenoma. **(A)**. image captured with FLUOBEAM® LX, **(B)**. Image captured with LX700, **(C)**. Image taken with LX700/775, **(D)** Region of interest (ROI) for the signal measurements based on the structure of the examined sample with the concentration and location of the indocyanine green (ICG) droplets and location of the parathyroid gland. 1, 0.05 μM ICG droplet; 2, 0.1 μM ICG droplet; 3, 0.25 μM ICG droplet; 4, 0.5 μM ICG droplet; 5, 1 μM ICG droplet; 6, Background for ICG droplets; 7, Parathyroid specimen; 8, Background for parathyroid specimen.

**Figure 4:**
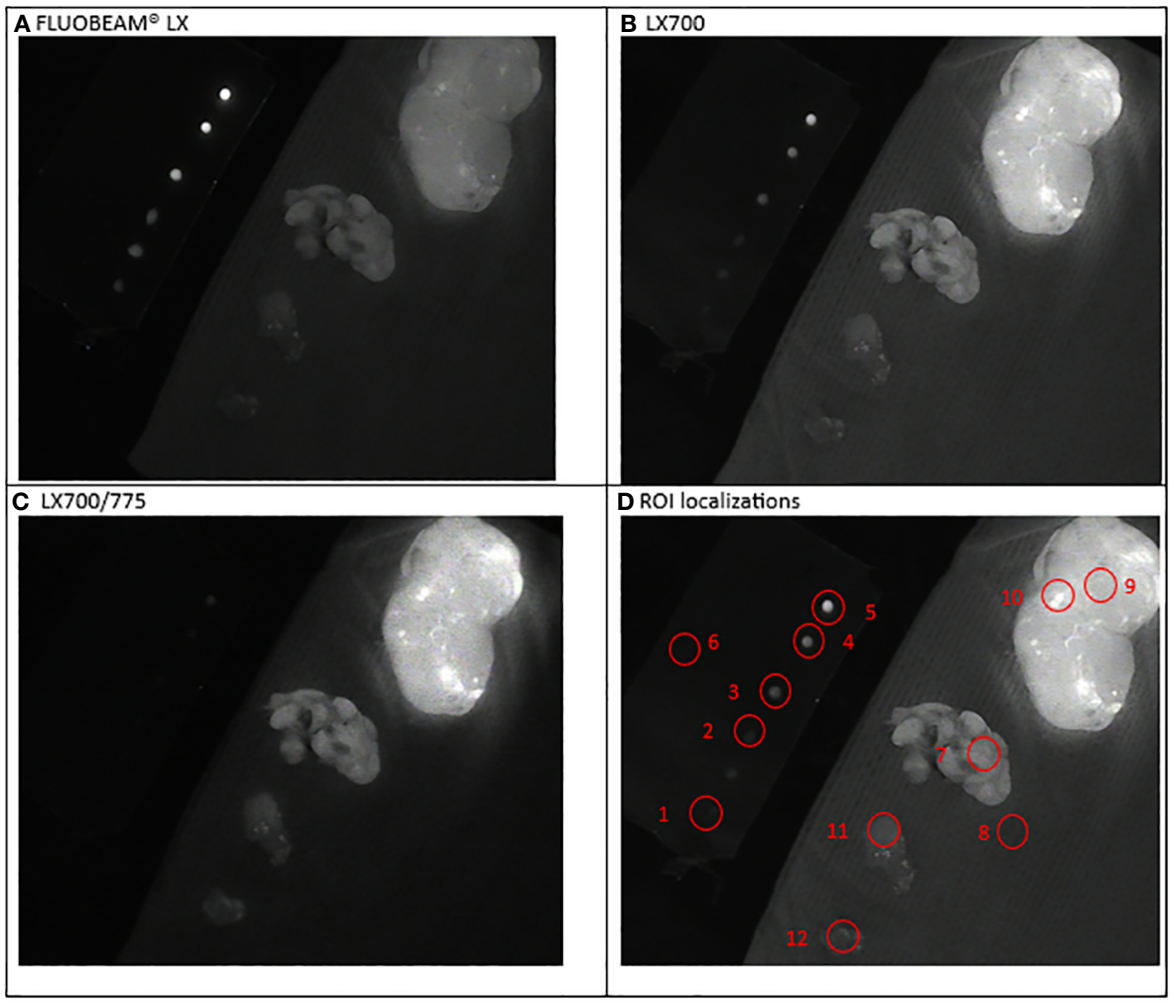
Second experimental setup: Parathyroid ablation. Simultaneous observation of the ICG droplets (top left corner) and specimen of neck tissues: extrathyroidal nodule with fluorescent grains (top right), parathyroid; nodule; brown fat (bottom left corner). **(A)** Image captured with FLUOBEAM® LX, **(B)** Image captured with LX700, **(C)** Image taken with LX700/775, **(D)** Region of interest (ROI) for the signal measurements based on the structure of the examined sample with the concentration and location of the indocyanine green (ICG) droplets and location of the parathyroid gland. 1, 0.05 μM ICG droplet; 2, 0.1 μM ICG droplet; 3, 0.25 μM ICG droplet; 4, 0.5 μM ICG droplet; 5, 1 μM ICG droplet; 6, Background for ICG droplets; 7, Parathyroid specimen; 8, Background for parathyroid specimen; For information only: 9, Thyroid nodule; 10, Thyroid nodule-fluorescent grain; 11, Nodule; 12, Brown fat.

**Figure 5:**
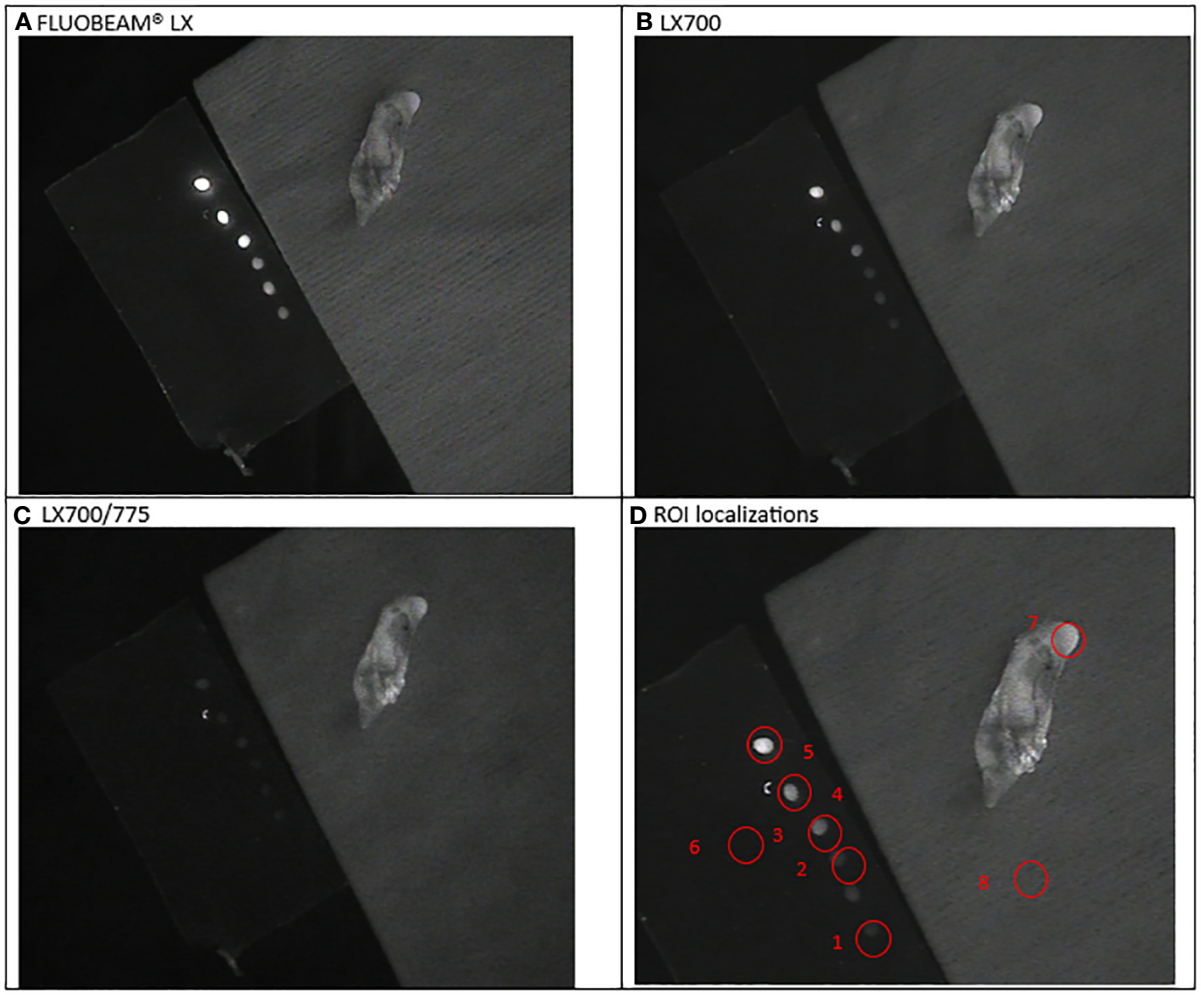
Third experimental setup: Parathyroid adenoma **(A)** image captured with FLUOBEAM® LX, **(B)** Image captured with LX700, **(C)** Image taken with LX700/775, **(D)** Region of interest (ROI) for the signal measurements based on the structure of the examined sample with the concentration and location of the indocyanine green (ICG) droplets and location of the parathyroid gland. 1, 0.05 μM ICG droplet; 2, 0.1 μM ICG droplet; 3, 0.25 μM ICG droplet; 4, 0.5 μM ICG droplet; 5, 1 μM ICG droplet; 6, Background for ICG droplets; 7, Parathyroid specimen; 8, Background for parathyroid specimen

**Figure 6:**
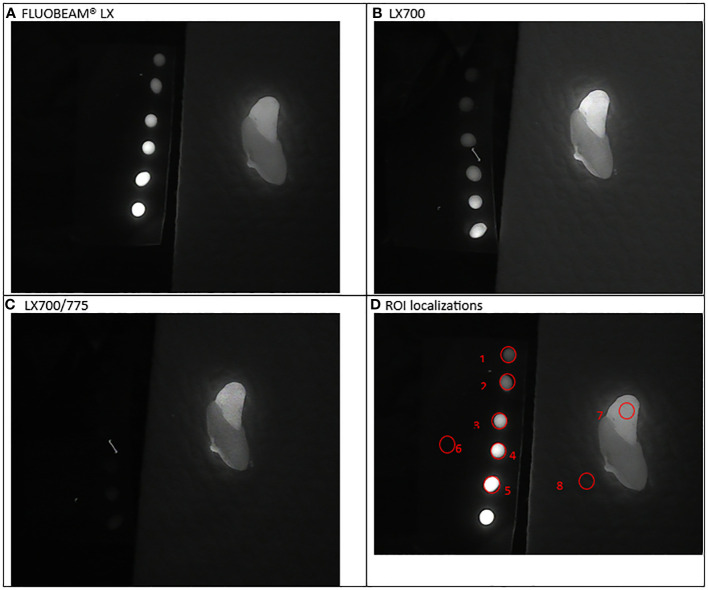
Fourth experimental setup: Parathyroid presenting a parathyroid nodule. **(A)** Image captured with FLUOBEAM® LX, **(B)** Image captured with LX700, **(C)** Image taken with LX700/775, **(D)** Region of interest (ROI) for the signal measurements based on the structure of the examined sample with the concentration and location of the indocyanine green (ICG) droplets and location of the parathyroid gland. 1, 0.05 μM ICG droplet; 2, 0.1 μM ICG droplet; 3, 0.25 μM ICG droplet; 4, 0.5 μM ICG droplet; 5, 1 μM ICG droplet; 6, Background for ICG droplets; 7, Parathyroid specimen; 8, Background for parathyroid specimen.

**Figure 7:**
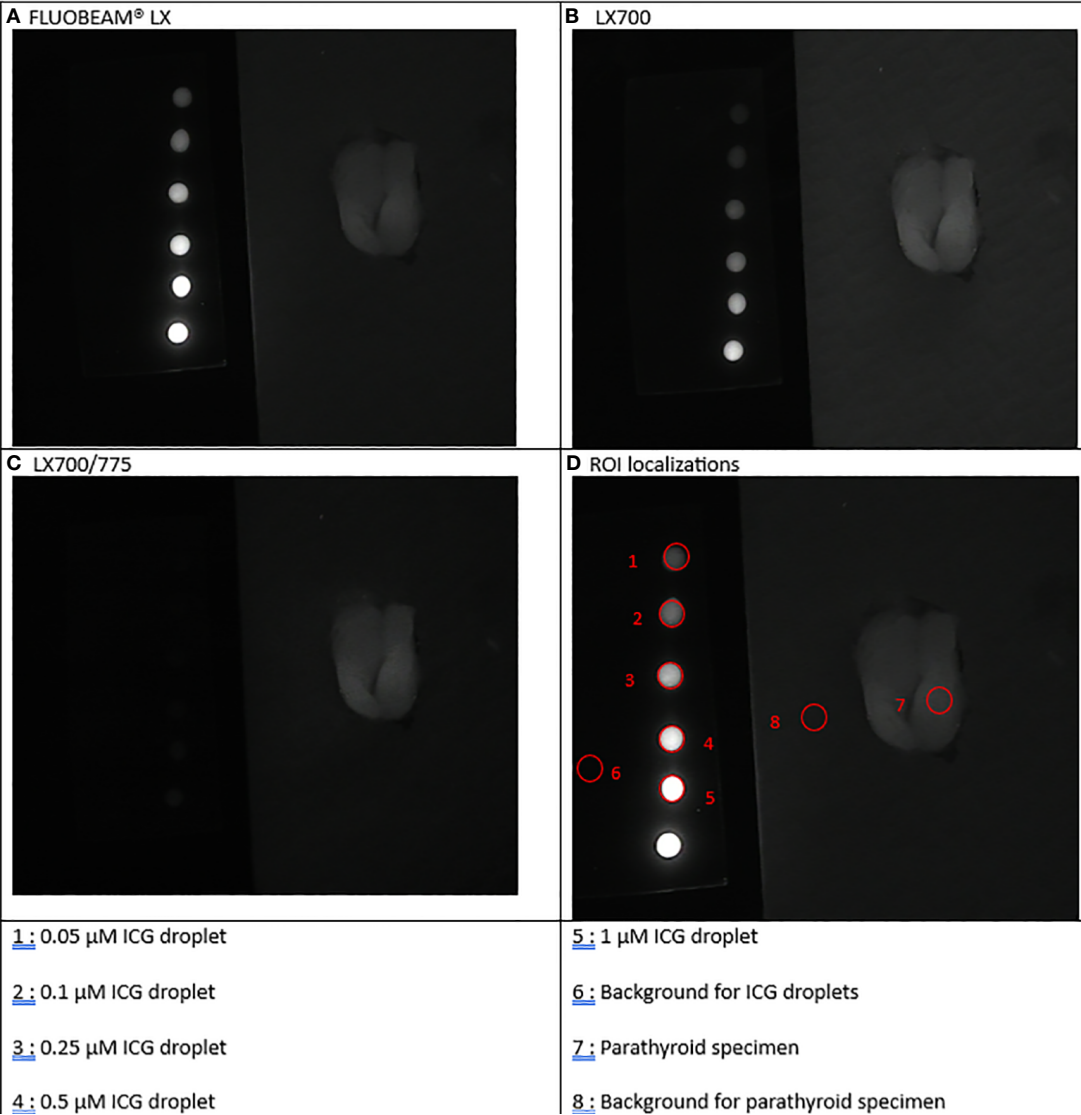
Fifth experimental setup: Parathyroid (split in two parts by the surgeon). **(A)** Image captured with FLUOBEAM® LX, **(B)** Image captured with LX700, **(C)** Image taken with LX700/775, **(D)** Region of interest (ROI) for the signal measurements based on the structure of the examined sample with the concentration and location of the indocyanine green (ICG) droplets and location of the parathyroid gland.

**Figure 8:**
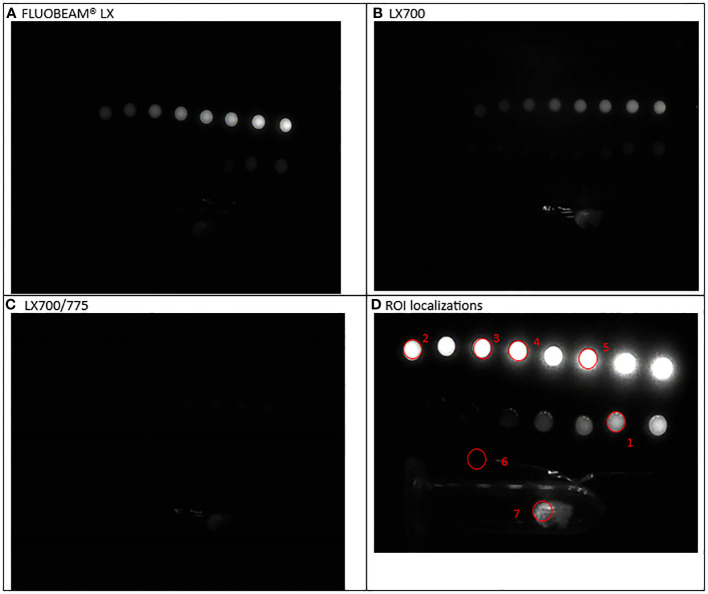
Sixth experimental setup: Parathyroid adenoma **(A)** Image captured with FLUOBEAM® LX, **(B)** Image captured with LX700, **(C)** Image taken with LX700/775, **(D)** Region of interest (ROI) for the signal measurements based on the structure of the examined sample with the concentration and location of the indocyanine green (ICG) droplets and location of the parathyroid gland. 1, 0.05 μM ICG droplet; 2, 0.1 μM ICG droplet; 3, 0.25 μM ICG droplet; 4, 0.5 μM ICG droplet; 5, 1 μM ICG droplet; 6, Background for ICG droplets and parathyroid specimen; 7, Parathyroid specimen.

To perform the tests, we used (i) five different dilutions of ICG, i.e., from 0.05 to 1 µM (0.05 µM, 0.1 µM, 0.25 µM, 0.5 µM, and 1 µM) in a solution containing 5% of albumin described earlier, which were arranged as drops of 10 µL, and (ii) freshly resected anatomical specimens of PTGs obtained during a parathyroidectomy. Six series of ICG droplets and resected PTGs were examined. The sample setup and areas of interest for each series are shown in **Figures 3**–**8**.

We measured the fluorescence signal using three possible system configurations, namely, FLUOBEAM_®_ LX, LX700, and LX700 with a low-pass filter at 775 nm, referred to as the LX700/775.

All acquisitions were performed under typical detection conditions for the PTGs with the FLUOBEAM_®_ LX, LX700, and LX700/775. The measurement distance was 10 cm, and the integration time was 40ms. Different camera amplification factors were used depending on the system configuration to provide useful, unsaturated images.

All results were scaled to a 40 ms/8.7 dB gain before computation. When the signal was saturated, the gain was reduced to obtain unsaturated data. Decreasing the camera gain does not impact the SNR.

### Data analyses

Data were extracted from digital images captured by the camera without any processing. Data extraction was performed using an image analysis software (ImageJ 1.53e). A region of interest (ROI) was designed for each droplet, and the mean values and standard deviations were computed for each ROI. Since the main variation of signal at pixel level was linked to camera amplification and smoothly varying fluorescence or autofluorescence levels in the ROIs, the signal distributions were almost symmetrical without outliers and could be characterized by means and standard deviations. An area without any samples was used as the background level reference. Signal distribution on these background areas was symmetrical without outliers because this signal is the reflectance of the remaining parasitic light by a uniform non fluorescent support. The following readings were obtained based on these formulas:

1) Signal measurement, S:


$$S=mean_{ROI}-mean_{background},$$

where $mean_{ROI}$ is the average value of the pixels of the ROI and $mean_{background}$ is the average value of the pixels in the area used as the background

2) Signal-to-noise ratio, SNR:


$$SNR=10*log_{10}\left(S/\sqrt{std_{ROI}^{2}-std_{background}^{2}}\right)$$

where $std_{ROI}$ is the standard deviation of the value of the pixels of the ROI and $std_{background}$ is the standard deviation of the value of the pixels in the area used as the background. The SNR was plotted using a logarithmic scale.

## Results

### Assessment of the PTG specimens and ICG droplets

The signals in the raw images provided by the LX700 were very similar to those acquired using the FLUOBEAM_®_ LX regarding their ability to detect ICG and autofluorescence. These raw images also showed that the LX700/775, with a short-pass filter at 775 nm, could visualize autofluorescence alone, even in the presence of high concentrations of ICG in the field.

**Figure 9:**
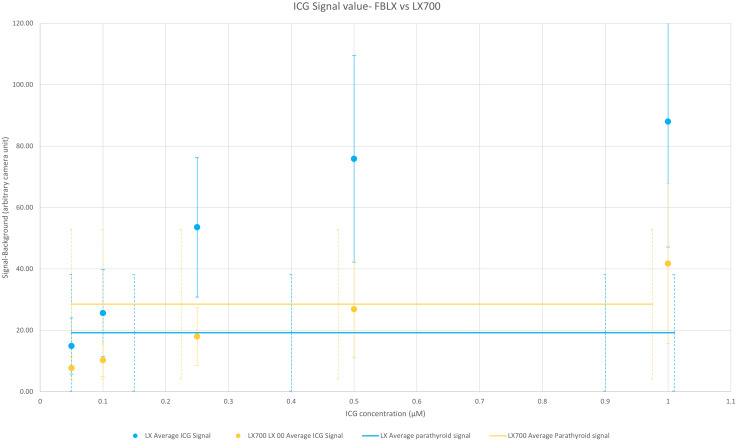
Comparison of the average value over the six setups of measured signals between the FLUOBEAM® LX and LX700 for the parathyroid gland (PTG) and indocyanine green (ICG) signals. For each data point, the subtracted background value was measured on the same dark area (an area where there was no sample). The range of the measured signal for the autofluorescence of the PTGs with FLUOBEAM® LX and LX700 is also indicated. This indicates that the same concentration of ICG could be easily detected by the two devices. The LX700 provides a signal almost 20% higher for the autofluorescence of the PTGs than the FLUOBEAM® LX.

To estimate the equivalence between the LX700 and FLUOBEAM_®_ LX, we compared the signal levels based on ICG and those regarding autofluorescence of the PTGs. **Figure 9** shows that the LX700 was more sensitive to PTG autofluorescence than FLUOBEAM_®_ LX. In contrast, the FLUOBEAM_®_ LX was more sensitive to ICG, which intrinsically produced very high signals.

**Figure 10:**
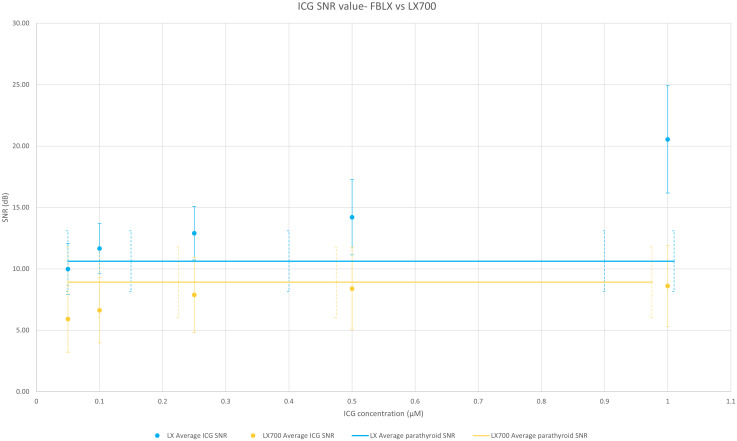
Comparison of the average value over the 6 setups of SNRs of the FLUOBEAM® LX and LX700 for a range of indocyanine green (ICG) concentrations. The two devices (FLUOBEAM® LX and LX700) could discriminate the same concentrations of ICG and were equivalent in terms of PTG detection. Even though the SNR was higher regarding ICG at low concentrations using the FLUOBEAM® LX, the SNR obtained using the LX700 stayed higher than 5 even for the lowest concentrations ensuring an unambiguous detectability.

To analyze the ability of these two systems to measure and discriminate signal variations, we compared the SNR of the two systems on the same samples. **Figure 10** shows that the SNR from the LX700 and FLUOBEAM_®_ LX were comparable for PTG signals and for the different concentrations of ICG. Even for low concentrations of ICG the SNR with the LX700 remained higher than 5dB ensuring unambiguous detectability.

**Figure 11:**
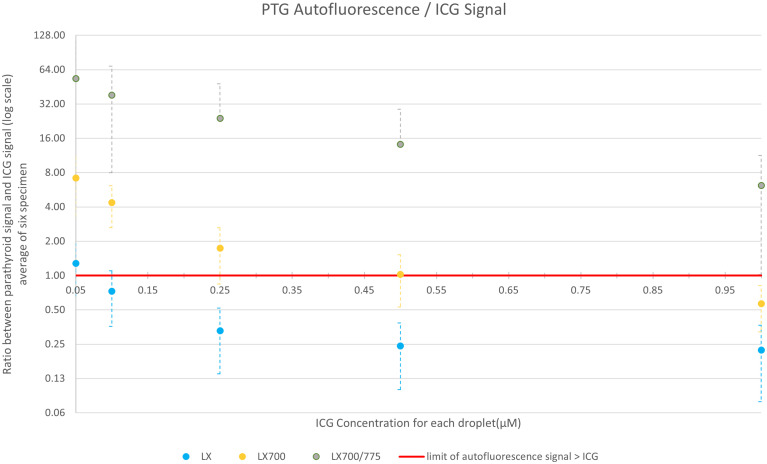
Comparison of the average value over the six setups of the ratio between ICG and parathyroid signals for the 3 systems: FLUOBEAM® LX, LX700 (unfiltered), and LX700/775 (low pass filtered; cut-off wavelength, 775 nm). The data used for this figure are presented in **Table 2**. Reducing the detection bandwidth with a low-pass filter decreased the overall sensitivity of the LX700/775 device. However, it increased its ability to discriminate parathyroid gland (PTG) autofluorescence in the presence of ICG by a factor of at least 60.

For fluorescence angiography using ICG, we compared these two devices with the LX700/775. Images were acquired with FLUOBEAM_®_ LX, LX700, and LX700/775. **Figure 11** shows the average ratio of the ICG signal (average signal value minus the background; the background was defined as an area with no ICG or PTG signal) and PTG signal for the five ICG concentrations listed in **Table 1**. Our results demonstrated that an additional 775 nm low-pass filter reduced the detected ICG fluorescence signal. Although the autofluorescence signal simultaneously decreased, the drop in the signal was limited; that is, for the highest ICG concentration (1.0 µM), the parathyroid signal was 7 times higher than the ICG signal (**Figure 11**). Therefore, it was possible to detect PTG autofluorescence in the presence of ICG using LX700/775 at ICG concentrations as high as 1.0 µM.

**Table 1:** Datasets regarding the three systems used in this study.

**Table 1.1:** FLUOBEAM® LX dataset.

	System	FLUOBEAM®LX							
	Set up #	1	2	3			4	5	6
	Initial gain	24.6	24.6	32.7			16.8	16.8	8.7
0.05 µM	Mean	10.93	8.04	11.60			31.98	31.06	14.20
	Stdev	1.25	0.88	0.96			1.70	1.20	1.47
0.1 µM	Mean	17.38	13.45	16.12			48.94	47.71	28.59
	Stdev	1.50	1.14	1.43			1.84	1.52	1.50
0.25 µM	Mean	38.55	36.26	31.05*			88.00	85.39	60.69
	Stdev	4.24	2.59	0.75			2.67	3.02	3.25
0.5 µM	Mean	55.99*	52.45	31.52*			119.63	117.65	96.42
	Stdev	4.54	4.12	0.17			2.46	2.86	5.44
1 µM	Mean	61.99*	62.03*	31.72*			123.76*	123.67*	143.16
	Stdev	0.60	0.41	0.10			0.25	0.21	7.91
Background for the ICG drops	Mean	3.50	2.02	3.09			4.40	3.54	1.99
	Stdev	0.20	0.14	0.35			0.27	0.26	0.11
Parathyroid	Mean	11.19	17.97	16.39			71.33	25.21	11.51
	Stdev	0.45	0.98	1.28			2.14	1.39	1.37
Background for the parathyroid	Mean	2.16	6.01	8.66			10.00	9.64	1.99
	Stdev	0.12	0.43	0.72			0.51	0.47	0.11

**Table 1.2:** LX700 dataset.

	System	LX700							
	Set up # #	1	2			3	4	5	6
	Initial gain	24.6	24.6			28.5	18.6	16.8	8.7
0.05 µM	Mean	11.37	7.30			6.87	9.46	11.93	10.82
	Stdev	0.98	0.65			0.54	0.47	0.56	1.35
0.1 µM	Mean	11.46	8.83			7.80	15.80	17.30	17.34
	Stdev	0.57	0.44			0.79	1.34	0.87	1.58
0.25 µM	Mean	17.99	15.96			16.79	27.33	31.23	36.98
	Stdev	1.58	1.08			1.25	1.29	1.86	2.31
0.5 µM	Mean	26.61	24.18			22.34	44.38	49.27	56.90
	Stdev	2.01	2.43			1.81	1.70	1.93	4.02
1 µM	Mean	43.08	41.37			37.17	80.80	79.39	83.66
	Stdev	4.77	3.31			3.23	3.51	2.83	6.58
Background for the ICG drops	Mean	7.82	5.11			4.41	3.83	4.73	2.17
	Stdev	0.68	0.32			0.30	0.28	0.36	0.38
Parathyroid	Mean	23.16	34.44			27.03	99.32	37.16	43.38
	Stdev	1.78	1.96			2.49	2.62	1.76	2.55
Background for the parathyroid	Mean	5.66	13.28			12.34	16.01	15.47	2.17
	Stdev	0.34	0.59			0.69	1.04	0.89	0.38

**Table 1.3:** LX700/775 dataset.

	System	LX700							
	Set up # #	1	2		3		4	5	6
	Initial gain	24.6	24.6		28.5		18.6	16.8	8.7
0.05 µM	Mean	11.37	7.30		6.87		9.46	11.93	10.82
	Stdev	0.98	0.65		0.54		0.47	0.56	1.35
0.1 µM	Mean	11.46	8.83		7.80		15.80	17.30	17.34
	Stdev	0.57	0.44		0.79		1.34	0.87	1.58
0.25 µM	Mean	17.99	15.96		16.79		27.33	31.23	36.98
	Stdev	1.58	1.08		1.25		1.29	1.86	2.31
0.5 µM	Mean	26.61	24.18		22.34		44.38	49.27	56.90
	Stdev	2.01	2.43		1.81		1.70	1.93	4.02
1 µM	Mean	43.08	41.37		37.17		80.80	79.39	83.66
	Stdev	4.77	3.31		3.23		3.51	2.83	6.58
Background for the ICG drops	Mean	7.82	5.11		4.41		3.83	4.73	2.17
	Stdev	0.68	0.32		0.30		0.28	0.36	0.38
Parathyroid	Mean	23.16	34.44		27.03		99.32	37.16	43.38
	Stdev	1.78	1.96		2.49		2.62	1.76	2.55
Background for the parathyroid	Mean	5.66	13.28		12.34		16.01	15.47	2.17
	Stdev	0.34	0.59		0.69		1.04	0.89	0.38

*Saturated signal

Tables 1.1, 1.2, and 1.3 show the measured values obtained from the analysis. ImageJ (software developed by NIH) was used to extract data on digitized fluorescence 8-bit images provided by the camera. For each droplet and each parathyroid gland region of interest, the averages (Mean) and standard deviations (Stdev) were computed with the following different acquisition configurations: FLUOBEAM® LX, LX700, and LX700 with 775 nm low-pass filter (referred to as the LX700/775). Different camera amplification factors were used depending on the system configuration to provide useful, unsaturated images. All results were normalized to a 40ms/8.7dB gain before computation.

**Figure 12:**
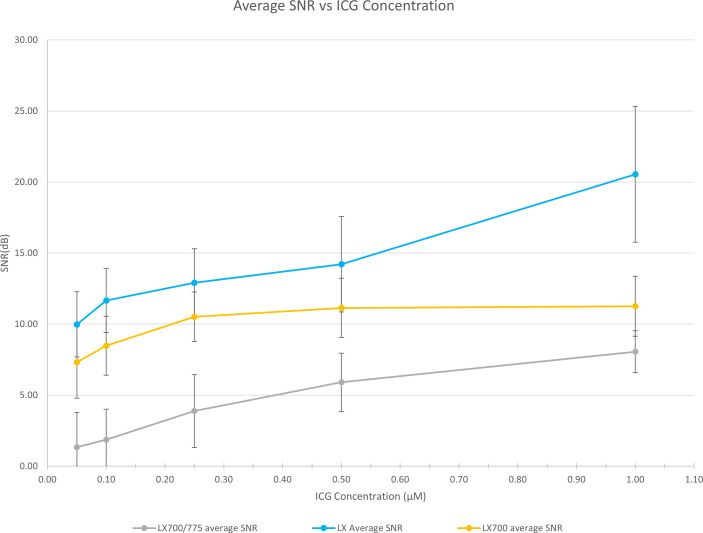
Comparison of the measured SNRs for the 3 systems (FLUOBEAM® LX, LX700, LX700/775). The low-pass filtering decreased the signal level by detecting on a narrower bandwidth. However, as the detection was performed on a narrower bandwidth, the background noise was significantly reduced.

To demonstrate that this type of approach does not worsen the detection quality of the autofluorescence of PTGs, we analyzed the SNR of the different configurations of the systems (FLUOBEAM_®_ LX, LX700, LX700/775) on the same sample (**Table 2**). For all configurations tested, the average SNR for the PTGs was close to 10, indicating that even with a reduced detection bandwidth of 700–775 nm (corresponding to the LX700/775 detection bandwidth), PTG autofluorescence could be unambiguously detected. **Figures 11**, **12** and **Table 2** show that the decrease in the SNR of the ICG droplets linked to the additional low-pass filter enabled the detection of PTG autofluorescence in the presence of ICG.

**Table 2:** SNR of Parathyroid glands with the three devices.

	LX	LX700	LX700/775
SNR (dB)	10,623	10,781	9,985
Stdev	2,476	2,190	1,695

The averaged SNR has been computed from the data of **Table 1**. Variation of the parathyroid gland (PTG) autofluorescence according to the filtering. The SNR was slightly lower with the LX700 filtered at 775 nm because only about 60% of the emitted spectra were detected. The SNR with the FLUOBEAM_®_ LX was comparable to that with the LX 700 because although only about 40% of the emitted autofluorescence signal was detected, the detection window was at a higher wavelength. Therefore, it was much less sensitive to background signals such as parasitic lights.

**Figure 13:**
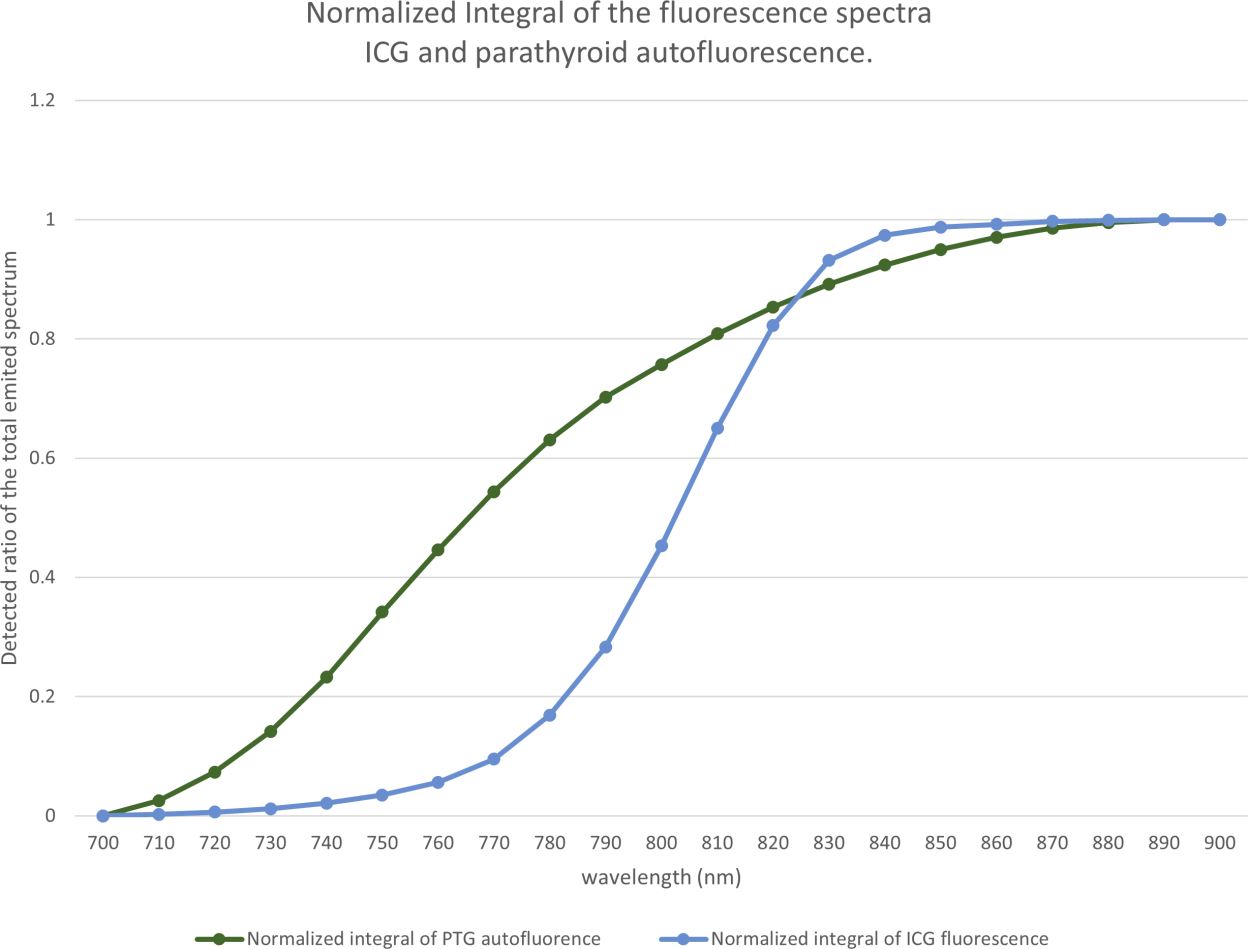
Proportion of the fluorescence signal as a function of the cut-off wavelength. Based on the spectra of **Figure 1**, for each wavelength between 700 and 900 nm, for indocyanine green (ICG) and the parathyroid gland (PTG), we computed the ratio of the spectra that would be detected by a device using a low-pass filter at a particular wavelength.

From the spectra in **Figure 1**, we calculated the measured signal for a low-pass filter with a specified cut-off length. This value correspond to the area below the curve in **Figure 1**, limited by the 700nm wavelength on one side and by the cut off wavelength on the other side. The proportion of the fluorescence signal, as a function of the cut-off wavelength, is shown in **Figure 13**.

The difference between the two curves allowed us to locate the maximum difference between the ICG and the autofluorescence signals near 775 nm. We observed that, with a low-pass filter at 775 nm, 60% of the autofluorescence signal and 10% of the ICG signal were detected. With a 750 nm filter, we detected 34% of the autofluorescence signal and 3% of the ICG signal.

### Other possible data that were processed using the two detections windows

**Figure 14:**
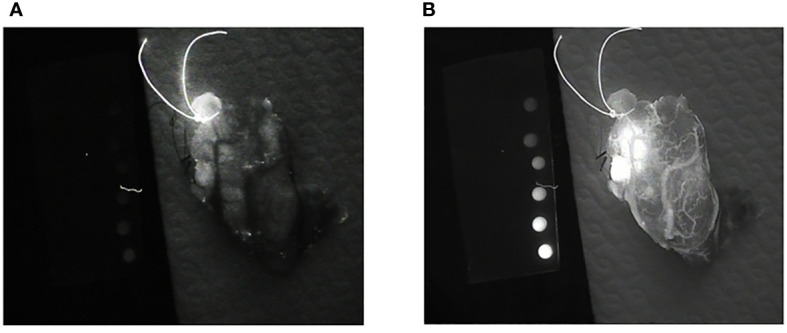
Images of the same thyroid lobe after excision. This lobe was removed after the injection of indocyanine green (ICG) to identify the blood vessels feeding the parathyroid glands. **(A)** Image obtained using a low-pass filter with a cut-off wavelength of 775 nm (LX700/775). **(B)** Image obtained with a nonfiltered device (LX700), in which the ICG contribution was clearly predominant.

An LX700 with a retractable low-pass filter at 775 nm can provide two images of the same sample containing different proportions of ICG and autofluorescence signals. The unfiltered image (LX700) was dominated by ICG, and the filtered image (LX700/775) was dominated by autofluorescence (**Figure 14**).

To be able to analyze the data provided by the device, we acquired two sets of two images. With the low-pass filter on (LX700/775), we acquired one image with the excitation laser off and one image with the excitation laser on. With the low-pass filter off (LX700), we acquired one image with the excitation laser off and another with the excitation laser on. For each position of the low pass filter, the difference between the measure with the laser on and off provided the actual contribution of the autofluorescence signal plus the ICG signal on the corresponding detection waveband.

Referring to the autofluorescence signal of the tissues emitted between 700 nm and 900 nm as AFsignal and the ICG signal emitted between 700 nm and 900 nm as ICGsignal , we can define α and β, two numbers between 0 and 1, such that α*AFsignal is the autofluorescence signal of tissues emitted between 700 nm and 775 nm and β*ICGsignal is the ICG signal emitted between 700 nm and 775 nm.

Then we can write that


Image with the filter off=AFsignal+ICG signal

Image with the filter on=α∗AFsignal+β∗ICG signal

If α and β have been measured in independent samples, we can easily produce images of ICG or autofluorescence alone.

We measured α on the set of thyroid and parathyroid specimens presented earlier and β on ICG droplets. We observed α=0.3 (for thyroid and parathyroids) and β=0.025.

In the first approach, assuming that the contribution of ICG represented by β*ICG is negligible when the low-pass filter is on, contributions of ICG and autofluorescence can be split to increase the accuracy of the provided information.

**Figure 15:**
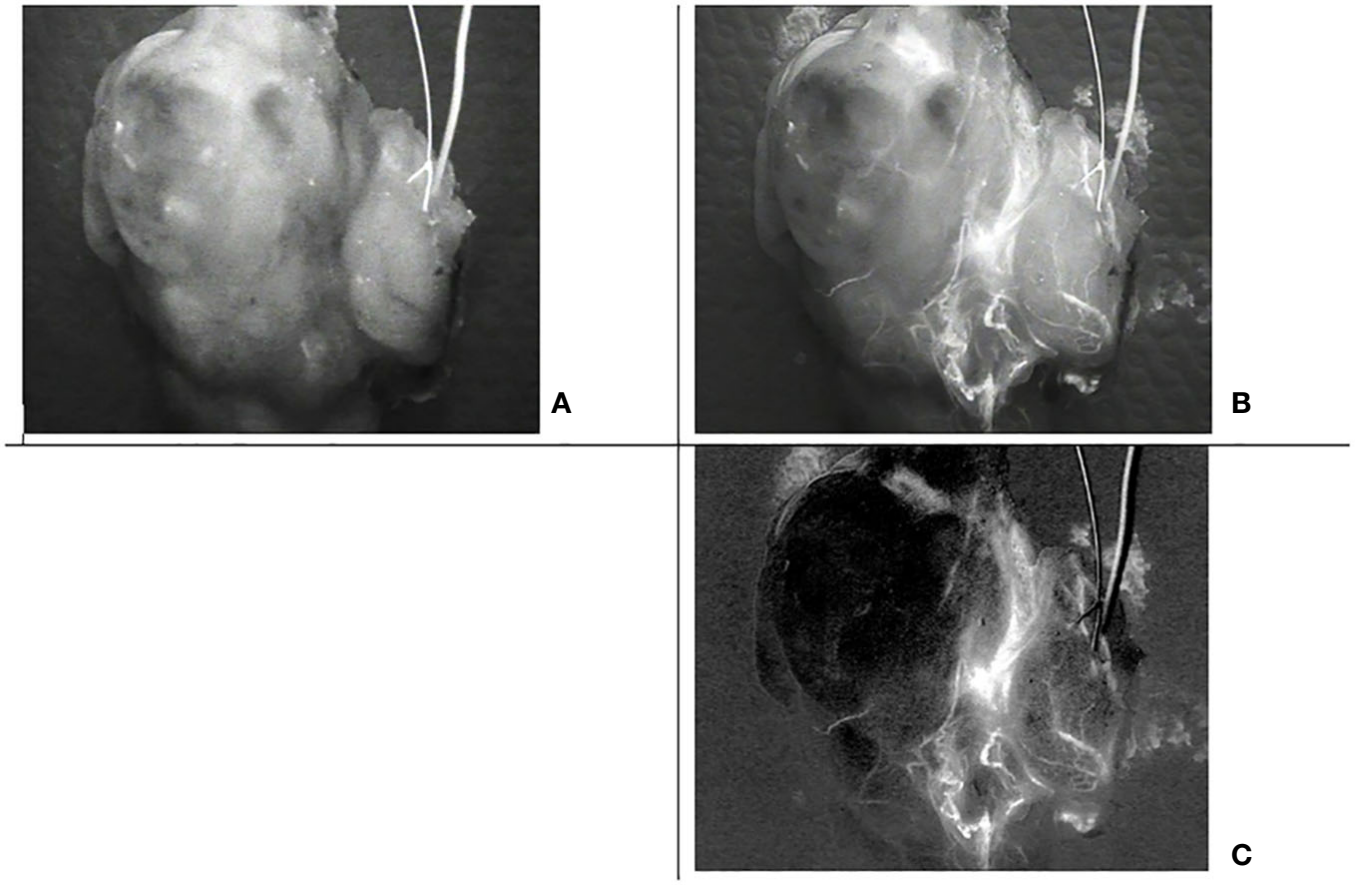
Images of the same thyroid lobe after excision. This lobe was removed after the injection of indocyanine green (ICG) to identify the blood vessels feeding the parathyroid glands **(A)** Image with the low pass filter on, **(B)** Image with the low pass filter off, **(C)** Indocyanine green (ICG) image computed from the four acquisitions assuming that alpha= 0.3 and beta =0.025.

Once processed by subtracting the background signal, these images lead to one image containing only the ICG contribution (**Figure 15**). Although the ICG image is noisier than the image with the filter off, it is not ambiguous in the perfused area, and small vessels can easily be discriminated.

## Discussion

Until recently, it was commonly accepted that the maximum autofluorescence of the PTGs is observed between 800 and 900 nm and that this autofluorescence is reduced significantly below 800 nm (3, 9, 12, 13). Thus, PTG autofluorescence used to be measured between 800 and 900 nm. Furthermore, as the excitation wavelength is between 740 and 805 nm in most commercially available systems, fluorescence is usually detected between 800 and 900 nm in these systems, e.g., PTeye (Medtronic), FLUOBEAM LX (Fluoptics), EleVision IR platform (Medtronic), and PDE (Hamamatsu). Balasubramanian et al. (14), using the FLUOBEAM® 700, indicated that PTG autofluorescence is visible with an excitation wavelength of 685 nm and an emission window between 700 and 900 nm. This autofluorescence allowed for the pre-localization of the PTGs during thyroidectomy. More recently, Serra et al. (10), using fluorescence spectrometry and excitation at 650 nm, confirmed that the PTGs emitted autofluorescence at least at 700 nm and that maximum autofluorescence was observed close to 711 nm.

In order to avoid using two excitation wavelengths, we examined a single excitation that rebalanced the measured signal levels between ICG and PTG autofluorescence. Exciting at a wavelength of 685 nm reduces the ICG contribution and increases the autofluorescence signal. Nevertheless, the contribution of PTG autofluorescence over a detection window ranging from 700 to 900 nm is negligible compared to that of ICG. Narrowing the detection window between 700 and 775 nm will reduce the contribution of ICG more than that of the PTG.

In our study, we observed that PTG autofluorescence could be visualized with a good SNR ratio upon excitation at 685 nm. Moreover, the autofluorescence signal was substantial between 700 and 800 nm.

We also demonstrated that the SNR of the autofluorescence signal obtained with an excitation at 685 nm and a detection between 700 and 775nm is similar to that obtained with the FLUOBEAM® LX (about 10 dB). This imaging approach opens new opportunities for the possibility of i) using autofluorescence alone even after injection of ICG and ii) combining images with different detection windows (700–900 nm and 700–775 nm) to split the contribution of ICG and autofluorescence.

By considering the possibility of renewing the ICG injections if necessary while observing the parathyroids by autofluorescence, the technical improvement described here removes the current main obstacle of intraoperative evaluation of the PTGs by fluorescence imaging at the end of the dissection of each lobe. Once the ICG has been injected, it would not be possible to observe the autofluorescence because the signal is dominated by ICG. However, identification by autofluorescence is crucial because it aids in PTG identification, which positively impacts the quality and function of the preserved glands (4).

By contrast, the injection, sometimes repeated, of ICG helps improve the dissection and/or intraoperative decision by helping to create a vascular mapping of the PTGs before thyroid dissection (8). It can also help improve the dissection by helping to evaluate the parathyroid perfusion at the end of the lobectomy, which would allow for the adaptation of the radicality of the thyroid resection on the second lobe in cases of total thyroidectomy (15). Both modalities are greatly facilitated by the possibility of easy and accurate visualization of the PTG with autofluorescent imaging during ICG injection.

Without the innovation described in this study, to be able to use autofluorescence on the four PTGs without endangering the recurrent nerve in case of loss of signal requiring a stop of the procedure and re-intervention in an already dissected area, the surgeon has to reserve mapping angiographies and perfusion assessment to one side only (the second) during total thyroidectomies.

In conclusion, this study demonstrates that PTG autofluorescence can be detected in the presence of ICG, in concentrations as high as 1.0 µM, with a fluorescence imaging device performing excitation at 685 nm and with detection between 700 and 775 nm. It also demonstrates that ICG can be detected at concentrations as low as 0.05 µM with the same excitation at 685 nm and a detection window between 700 and 900 nm.

The expected clinical benefits versus drawbacks (added time, expense, etc.) from using fluorescence-based parathyroid imaging combined with ICG angiography compared to either technique alone or other imaging methods must be evaluated because the potential for this combination is the key advance offered by changing the excitation and emission settings.

This feasibility study needs to be complemented by an extensive clinical assessment to evaluate the clinical benefits of using a device with excitation at 685 nm and a removable low-pass filter for different pathologies leading to thyroid and/or parathyroid surgery.

## Data availability statement

The original contributions presented in the study are included in the article/supplementary material. Further inquiries can be directed to the corresponding author.

## Author contributions

MR and PR contributed equally to the conception, design, and data analysis of the study. All authors contributed to the article and approved the submitted version.
